# The contemporary challenge for ethical research involving the knowledge of indigenous peoples and local communities and afro-descendants and other marginalized, minority, or minoritized groups

**DOI:** 10.1186/s13002-025-00774-4

**Published:** 2025-04-26

**Authors:** Ulysses Paulino Albuquerque, Romulo Romeu Nóbrega Alves, Washington Soares Ferreira Júnior

**Affiliations:** 1 Laboratório de Ecologia e Evolução de Sistemas Socioecológicos, Departamento de Botânica, Centro de Biociências, Universidade Federal de Pernambuco (UFPE), Recife, PE 50670-901 Brazil; 2 Laboratório de Etnobiologia, Universidade Estadual da Paraíba (UEPB), Bairro Universitário, Avenida das Baraúnas, 351, Campina Grande, PB 58429-500 Brazil; 3 Laboratório de Estudos Etnobiológicos, Universidade de Pernambuco (UPE), R. Amaro Maltês de Farias, Nazaré da Mata, PE 55800-000 Brazil

**Keywords:** Ethnobiology, Epistemic justice, Ethical research, Indigenous systems of knowledge, Knowledge co-production

## Abstract

The publication of ethnobiological data raises crucial ethical questions regarding the rights of Indigenous Peoples and Local Communities (IPLC) and Afro-descendants and other Marginalized, Minority, or Minoritized Communities (AMMC). While ethnobiology as a discipline is rooted in ethical principles that emphasize respect and appreciation for these communities, the question remains: Is publishing ethnobiological data always respectful of knowledge holders’ rights? This article argues that the answer is contingent on how research is conducted, how consent is obtained, and how data is handled and disseminated. We emphasize the need for a nuanced approach that goes beyond compliance with ethical guidelines and embraces the principles of epistemic justice, equitable benefit-sharing, and genuine co-production of knowledge. By distinguishing between raw traditional knowledge and ethnobiological data systematized within scientific paradigms, we highlight the potential risks of knowledge misappropriation and the epistemological implications of translating diverse knowledge systems into western scientific frameworks. We also discuss the limitations of Free, Prior, and Informed Consent (FPIC) as a safeguard and propose alternative strategies for ensuring IPLC and AMMC autonomy in the knowledge production process. Finally, we advocate for hybrid co-production of knowledge as a transformative approach to fostering equitable collaborations between researchers and communities. By embedding ethical considerations at every stage of the research process, we argue that ethnobiology can evolve into a discipline that actively contributes to social justice, sustainability, and the recognition of diverse epistemologies.

## Introduction

The question, *“Is Publishing Ethnobiology Data Respectful of Indigenous and Local Knowledge Holders’ Rights?”* prompts a central and urgent debate within ethnobiology. Any researcher familiar with the historical trajectory of this discipline, which is deeply rooted in ethical principles of respect and appreciation for the rights of Indigenous peoples and local communities, would recognize that this question cannot be answered simply. A direct “yes,” devoid of broader reflections on intellectual property, informed consent, and epistemic justice, would not only be misguided but would also contradict the very pillars of contemporary ethnobiological practice, as repeatedly emphasized by numerous researchers [1–5].

Nevertheless, it is conceivable to arrive at an ethically legitimate and responsible “yes”—provided that rigorous principles of respect and appreciation for the rights of these communities guide it. This affirmative answer depends on a series of conditions that ensure that data publication is informed, culturally sensitive, and aligned with the aspirations and realities of the populations involved. Therefore, the most appropriate answer to this question is not a simple “yes” or “no” but an emphatic *“it depends.”*

This article adopts this conditional and nuanced perspective to explore the issue’s complexities. The publication of ethnobiological data can indeed be conducted in a way that respects Indigenous and local knowledge holders’ rights. However, this requires more than mere adherence to basic ethical standards. It necessitates practices that transcend superficial compliance and instead embrace the richness and diversity of the rights, knowledge, and interests of the communities that hold such knowledge.

To substantiate our position, we present arguments illustrating how data publication can simultaneously serve as a powerful tool for valuing Indigenous and local knowledge and as an ethical process that safeguards the rights of its holders (see [6–8]). Throughout this discussion, we assert that the challenge is not merely about deciding whether to publish but about determining how to publish to advance justice, epistemic equity, and mutual appreciation.

A critical starting point for any philosophical or epistemological discussion on publishing ethnobiological data is recognizing that the communities’ power to decide what can and cannot be published rests fundamentally. Such decisions must consider cultural contexts, the potential risks of disclosing sensitive data, and the intrinsic value of certain knowledge. Consequently, it is essential to establish an ethical pact that restores and reinforces communities’ agency, ensuring they retain control over the narrative surrounding their knowledge.

Our approach in this article will be expressly limited to Indigenous Peoples and Local Communities (IPLC), as well as Afro-descendants and Afro-descendants and other Marginalized, Minority, or Minoritized Communities (AMMC). We use the term underrepresented communities or IPLC and AMMC to simplify references throughout the text.

## What kind of ethnobiological data are we referring to?

When we refer to ethnobiological data, we are addressing something very specific: knowledge that, while it may originate from traditional ecological knowledge (TEK) or local ecological knowledge (LEK), has been collected, organized, systematized, and interpreted within a scientific framework. In other words, these data result from an intellectual process in which TEK/LEK —whether sensitive or not—is transformed into information that aligns with scientific paradigms and epistemological criteria. Ethnobiological data can range from the description of knowledge systems that emerge from human-biota interactions, such as fundamental characteristics of these systems (including the popularity and versatility of local resources, redundancy, and pathways of biocultural knowledge transmission) to parameters that reveal the factors explaining their structure, dynamics, and evolution. These systems can also incorporate sacred and mystical dimensions, reflecting cultural, spiritual, and symbolic perceptions that guide communities’ relationships with nature. By examining both tangible (e.g., resource use patterns, redundancy) and intangible (e.g., sacred and mystical dimensions) aspects of knowledge systems, researchers can better grasp the complexity of biocultural interactions.

This organized data is fundamentally different from raw data. Raw data may consist of elements of biodiversity or cultural practices as they are understood and interpreted through TEK/LEK. Such data are intrinsically tied to the sociocultural contexts in which they emerge, often carrying meanings that transcend the boundaries of scientific interpretation. For example, a community’s medicinal plant use might be embedded in a system of spiritual beliefs or local management practices—dimensions that may not be fully captured or conveyed when raw data is systematized into scientific formats. A single ethnobiological study may integrate both types of data. For instance, research conducted from an emic perspective, which seeks to assess local perceptions of specific aspects of nature, can incorporate raw data (TEK/LEK and interpretations) while also integrating scientific analyses that ultimately generate ethnobiological data.

It is also important to clarify that our understanding of ethnobiology extends beyond traditional or local knowledge of IPLC and AMMC. While such knowledge forms a cornerstone of the field, ethnobiology, as we define it, is the scientific study of the coevolution between humans and nature [9, 10]. This perspective broadens the scope of ethnobiological data by considering not only the systematization of traditional ecological knowledge and other forms of human-nature interaction mediated by cognitive, historical, social, and ecological processes that may or may not be directly associated with IPLC and AMMC.

Interactions between people and biota are highly diverse and can be explored through disciplines spanning the social and natural sciences. These fields examine the historical, economic, sociological, anthropological, and environmental dimensions of human-nature relationships. Consequently, ethnobiology integrates perspectives from biologists, anthropologists, sociologists, historians, geographers, pharmacologists, ecologists, archaeologists, and political scientists, among others. This interdisciplinary approach is essential given the complexity of these interactions.

Ethnobiological data are valuable not only to the biological sciences but also to a broad range of disciplines, enhancing our understanding of ecological, cultural, and social dynamics. For instance, knowledge of plants and animals provides crucial insights into how humans distribute and utilize natural resources across different regions. Studies on land occupation, territorial management, and sustainable resource use contribute to ecological research while also informing geographic, anthropological, and conservation perspectives.

Different academic fields can have different ethical codes, as ethical guidelines are often shaped by the specific nature of research, the subjects involved, and the potential impacts of the studies. Because of these differences, ethnobiological researchers must navigate multiple ethical frameworks, ensuring respect for both human communities and ecological systems.

Thus, when discussing ethnobiological data, we refer to information that emerges from a scientific epistemology, acknowledging that the process of systematization is an interpretative act shaped by the scientific context in which it occurs. This perspective broadens the scope of ethnobiological analysis, enabling it to address more extensive and complex phenomena beyond the mere documentation or valorization of TEK/LEK. At the same time, it underscores science’s ethical responsibilities and limitations when engaging with such data. Ethnobiological data, therefore, reflect not only the original knowledge but also the choices, priorities, and assumptions inherent in the scientific system that organizes and interprets it.

## Safeguarding rights

It may seem surprising to write, in 2025, about the importance of safeguarding rights and building trust with IPLC and AMMC in ethnobiology. After all, this is not a new issue but a core principle that dates to the earliest ethical frameworks of the discipline. The explanation for this apparent anachronism is straightforward: Darrell Posey, one of the pioneers of ethnobiology, raised these issues as early as the 1980s, if not earlier, emphasizing the critical need to respect the rights and agency of these underrepresented communities in the production and use of TEK/LEK.

Similarly, it may seem redundant to reiterate the significance of Indigenous and local knowledge, as one might expect that, as an academic community, we would have moved beyond this foundational stage of the debate. Today, philosophical and epistemological discussions in the field have advanced considerably, addressing topics such as epistemic justice, equitable benefit-sharing, and the complex interplay between diverse knowledge systems (see [11–13]. However, the need to return to these fundamentals—safeguarding rights and building trust—remains pressing because, in practice, significant gaps still exist in applying these foundational principles. These challenges extend beyond the academic realm, as political and economic pressures on IPLC and AMMC and their territories play a significant role, mainly in countries with far-right government.

Adherence to international protocols regulating access to traditional knowledge is central to the publication of ethnobiological data. Instruments such as the Convention on Biological Diversity (CBD), the Nagoya Protocol, and the United Nations Declaration on the Rights of Indigenous Peoples emphasize the right of Indigenous and local peoples to control the use of their knowledge and resources. These documents establish clear guidelines to ensure that access to and dissemination of traditional knowledge respects the autonomy, interests, and cultural rights of IPLC and AMMC.

Moreover, these protocols remind us that free, prior, and informed consent is not merely an ethical obligation but an essential safeguard against the misappropriation of knowledge. They ensure that IPLC and AMMC retain agency over decisions that affect them, reinforcing their role as key stakeholders in managing and disseminating their knowledge.

In some countries, discussions surrounding safeguarding rights and intellectual property in ethnobiological research have yet to reach full maturity. Despite this, the publication of ethnobiological data continues unabated. However, this does not mean such publications are free from ethical concerns. On the contrary, in some instances, the data published are sensitive and, without a doubt, compromise the rights of the communities involved. This directly contravenes principles that protect intellectual property and respect knowledge holders.

Institutional barriers to the implementation of ethical protocols in ethnobiology research highlight challenges that extend beyond mere regulatory compliance. As discussed by us in a collaborative editorial involving editors from several scientific journals [14], the rigidity of Institutional Review Boards (IRBs) can conflict with culturally appropriate methodologies, particularly in studies conducted with IPLC and AMMC. While these committees play a crucial role in protecting research participants, their lack of flexibility can lead to prolonged bureaucratic processes and, in some cases, hinder essential research. Additionally, the absence of formal ethical review mechanisms in certain non-academic institutions exacerbates the issue, leaving researchers without clear guidelines to ensure ethically responsible studies [14].

Under such circumstances, it is impossible to overlook the risks of publishing information without thoroughly considering the ethical and legal implications. Sensitive data—such as traditional knowledge about the use of natural resources, medicinal practices, or cultural beliefs—can be misused by third parties, leading to unauthorized commercial exploitation, marginalization of underrepresented communities, or even the misappropriation of knowledge. Regardless of the format or justification, publishing such information without proper safeguards is profoundly disrespectful and can cause significant harm.

In these cases, the answer to whether it is legitimate or ethical to publish such data must be an emphatic and unequivocal *No, it is not respectful.* The lack of robust discussions or comprehensive policies in certain countries cannot be used to justify neglecting the rights of communities. On the contrary, these gaps make it even more imperative for researchers to adopt a rigorous ethical stance. They are responsible for ensuring that no publication compromises the rights or interests of the populations that have entrusted their knowledge to research.

The solution to addressing ethical weaknesses related to the publication of sensitive ethnobiological data lies in establishing a collective pact among all actors involved in the scientific production process: researchers, editors, reviewers, and authors. This pact must foster a transparent and non-negotiable commitment to reject publishing articles that fail to meet minimum ethical standards, ensuring that scientific advancement does not compromise the rights, dignity, and interests of IPLC and AMMC.

As the starting point, researchers are responsible for conducting their studies ethically, prioritizing protecting community rights from the initial planning stages to disseminating results. Ethnobiological researchers must adhere to the discipline’s ethical codes and go beyond mere compliance with established standards by engaging in genuine and collaborative dialogue with the communities involved (e.g., [15]). Authors, likewise, must adopt an unwavering ethical stance, committing themselves to respecting the rights of the communities they work with and ensuring proper recognition and protection for all involved (see [14]). They must also resist the pressures of academic productivity that often prioritize quick results over ethical integrity.

In this context, editors and reviewers hold critical roles as custodians of scientific ethics. Scientific journals must implement rigorous policies requiring proof of adherence to ethical standards before accepting manuscripts for publication. Reviewers, in turn, should evaluate the scientific merit of a submission and its compliance with ethical requirements, refusing to recommend the publication of work that fails to meet these criteria.

When safeguarding rights and building trust are embedded and validated as integral parts of the editorial process, publishing ethnobiological data can become a powerful tool for valuing traditional knowledge. This approach expands the recognition of such knowledge and ensures its use respectfully and sustainably. The ultimate challenge is not merely to produce publications but to foster partnerships that embody the principles of equity and respect, advancing both the practical application and the critical reflection that guides the field of ethnobiology.

Some argue that Free, Prior, and Informed Consent (FPIC) is a powerful tool for ensuring Indigenous peoples’ and local communities’ sovereignty and decision-making power. This claim holds merit pragmatically, as FPIC establishes an ethical and legal foundation for engaging with these communities. However, when viewed through the lens of building genuine safeguards for rights and fostering trust, the practical application of FPIC reveals significant limitations.

While FPIC is widely recognized as a cornerstone of ethical ethnobiological research, our experience conducting studies in the semiarid region of northeastern Brazil highlights a critical issue: even after signing consent forms, many participants did not fully understand the objectives of the research or the implications of their participation This underscores a fundamental problem: simply obtaining consent does not guarantee that it is fully informed or that communities are genuinely aware of the potential risks and benefits involved.

In such cases, providing consent often does not equate to a comprehensive understanding of the research and its consequences, particularly in contexts where participants have limited access to clear, culturally adapted information. This disconnects challenges the effectiveness of FPIC as a tool for fostering true agency and emphasizes the need for more inclusive, transparent, and culturally sensitive communication strategies in research practices.

## Epistemic justice and recognition

When conducted with respect and responsibility, the publication of ethnobiological data transcends mere academic acts—it becomes a practical expression of epistemic justice. This concept calls for the voices and knowledge of historically marginalized groups to be recognized and treated equitably on a global stage. Ignoring or marginalizing Indigenous and local knowledge perpetuates *epistemicide*—the systematic destruction of knowledge systems and cultural practices associated with specific communities. Such neglect is profoundly unjust and robs humanity of diverse perspectives vital for addressing global challenges like climate change and biodiversity loss.

However, aligning with the broader, contemporary movement of decolonization—which extends beyond ethnobiology and has gained traction worldwide—requires more than acknowledging knowledge (see [15, 16]). The communities with this knowledge must build, shape, and expand it alongside researchers. This collaborative approach not only enriches the field of ethnobiology but also empowers communities by fostering autonomy, cultural pride, and self-determination. In this scenario, researchers working with IPLC and AMMC have been crucial, particularly in the recognition and maintenance of these communities’ territories in the face of various threats and pressures from external economic groups.

Such joint construction can be realized through various means. One of the most effective is integrating members of IPLC and AMMC as active participants in interdisciplinary research teams. In this model, knowledge holders contribute as co-creators and interpreters of discoveries, enabling a genuine exchange between traditional and scientific knowledge systems (e.g., [16]). This approach fosters synergy between these systems while respecting each’s uniqueness.

Among the proposals to advance epistemic justice, including community knowledge holders as co-authors in scientific publications have emerged as a strategy for recognizing and valuing their contributions. While this practice is legitimate and commendable in many cases, it demands deeper consideration of its ethical and epistemological implications. Co-authorship in scientific articles is not a neutral act—it entails the tacit acceptance of a specific framework for understanding and describing the world, shaped by the norms that govern the production, validation, and dissemination of knowledge within science.

Academic knowledge operates within a particular epistemology, grounded in criteria such as objectivity, replicability, and universality, which can differ radically from traditional peoples’ epistemologies. For many IPLC and AMMC, knowledge is deeply embedded in cultural and spiritual practices, inseparable from lived experiences and oral traditions. Including these knowledge holders as co-authors may inadvertently impose a form of epistemology inconsistent with how their knowledge is traditionally produced, transmitted, and interpreted.

Moreover, scientific authorship comes with specific responsibilities, such as approving the final text and agreeing with the interpretations and conclusions presented. It is not always feasible or appropriate for community members to assume these responsibilities, especially when the shared knowledge is collective rather than individual. In some cases, attempts to include communities as co-authors may serve more to frame their epistemologies within dominant scientific paradigms than to foster genuine, equal cooperation.

By ethically and collaboratively publishing data, ethnobiologists can counteract centuries of invisibilization and marginalization of IPLC and AMMC systems of knowledge. Recognizing the critical role of knowledge-holding communities is a moral obligation and a vital contribution to sustainability and environmental conservation. However, this endeavor requires more than good intentions. It necessitates a deep commitment to epistemic justice and the creation of partnerships that respect diverse ways of producing and interpreting knowledge.

For example, the implementation of epistemic justice, equitable benefit-sharing, and knowledge co-production has been practically addressed through initiatives in political ethnobiology in Brazil [17, 18]. Soldati and Almada [17] highlight how the collaboration between researchers and traditional communities has led to concrete advancements, such as the development of popular dossiers to safeguard rights related to genetic heritage and traditional knowledge, as well as the creation of educational spaces aimed at political capacity-building for these communities. These efforts not only reinforce the autonomy of IPLC in defending their rights but also challenge the historical power asymmetries imposed by the dominant scientific model. A notable example is the partnership with the “Flowers Gatherers”, in which ecological research was employed to contest the criminalization of traditional harvesting practices, demonstrating that local community management of these species does not pose a threat to biodiversity [17].

Philosophically, ethical and collaborative publishing aligns with values of equity, respect for cultural diversity, and the preservation of multiple ways of being and understanding the world. However, it is essential to critically reflect on how such collaboration is implemented in practice. Are IPLC and AMMC genuinely included as protagonists in the scientific process, or are they being subsumed into dominant Western epistemological structures? Collaboration cannot be reduced to mere formalism or symbolic gestures. It must be built upon ongoing dialogue, transparency, and the recognition of traditional epistemologies (e.g., [19–21]).

An ideal and enriching scenario is when indigenous or local scientists produce knowledge about their realities, integrating lived experiences and traditional knowledge with scientific tools and methodologies (see [22]). This approach promotes epistemic diversity and offers an internal perspective closely attuned to the cultural, social, and environmental dynamics shaping these contexts. However, while such production is indispensable, it must not be used to exclude “non-native” scientists from engaging in ethnobiological research or other fields involving traditional knowledge.

The notion that only “native” scientists should conduct research in certain contexts risks creating an isolationist framework that could stifle interdisciplinary and intercultural dialogue. Additionally, it overlooks the history of science as a collective and collaborative endeavor that thrives on the convergence of diverse perspectives, experiences, and knowledge systems. Denying external scientists, the opportunity to engage with local communities may inadvertently reinforce barriers, limiting access to resources, collaborations, and global discussions.

Therefore, the challenge is not to exclude or delegitimize “non-native” scientists but to establish conditions where all participants in the knowledge production process—local or external—act ethically, equitably, and in true partnership. This inclusive approach enables scientific advancement without compromising communities’ rights, dignity, and autonomy, fostering a transformative research model. In this framework, the engagement of external scientists should not be perceived as a threat but as an opportunity to enrich collective knowledge and strengthen epistemic justice.

## Co-production of knowledge

By our definition, the co-production of knowledge is a hybrid process in which scientific and traditional epistemologies coexist and collaborate to generate something new. This hybridization goes beyond the mere combination of elements from each system in a scientific article or the translation of TEK/LEK into the frameworks of western science. Instead, it emerges when representatives of different knowledge systems engage as equals to address shared problems and challenges without subordinating one system to the logic or structure of the other.

This hybrid process is not a simple aggregation of perspectives; it entails creating a shared epistemic space where the contributions of each system are equally valued. Within this space, there is no need to “domesticate” or reshape TEK/LEK to fit scientific paradigms. Hybrid co-production instead seeks to forge new ways of understanding and acting, which can only arise from deep, ongoing dialogue between these different knowledge systems.

For instance, in tackling complex issues such as climate change, food security, or biodiversity conservation, TEK/LEK often provides contextual and practical insights that conventional science cannot achieve alone. Examples include local knowledge of weather patterns, sustainable resource management practices, or community governance systems. Conversely, science contributes analytical tools, technologies, and methodologies that can extend the applicability and impact of local knowledge. When these systems meet without imposed hierarchies, truly hybrid approaches emerge, combining the strengths of both.

The unique aspect of this hybridization is that it is not confined to a specific format, such as a scientific article or a traditional oral narrative. Instead, it manifests in the practices, solutions, and processes born from this collaboration—creating a third way that belongs neither entirely to science nor to TEK/LEK. This co-creation process acknowledges the contributions of all participants as full intellectual agents, each bringing their unique, yet equally essential, role to the table.

Rather than forcing integration or translating one knowledge system into the terms of another, hybrid co-production respects the integrity of both systems, allowing each to remain rooted in its logic while contributing to a shared endeavor. This approach promotes epistemic justice and points to a future where the diversity of ways of knowing and interpreting the world is recognized as an indispensable asset for addressing global challenges. The true potential of hybrid co-production lies in the realization that innovation and transformation emerge from the collaboration of diverse epistemologies without reducing or subordinating any of them.

Moreover, valuing epistemic diversity benefits IPLC and AMMC and strengthens science, making it more sensitive, relevant, and effective socially (see [23]). Publishing ethnobiological data due to a co-production process is a matter of justice and a scientific strategy to broaden understanding of global issues and foster sustainable solutions. By practicing co-production, researchers and communities jointly assume the role of transformative agents, pushing the boundaries of knowledge for the benefit of all.

## Final reflections

When we reflect on the question, *“Is Publishing Ethnobiology Data Respectful of Indigenous and Local Knowledge Holders’ Rights?”* it becomes clear that ethnobiological data are not merely elements collected directly from communities. Instead, they represent knowledge that undergoes an intellectual transformation shaped by specific epistemological frameworks when systematized and interpreted through scientific processes. While necessary to meet science paradigms, this transformation is far from neutral and carries profound ethical and epistemological implications.

Raw data, in its original form, is deeply connected to the cultural and spiritual meanings of the communities from which it originates. This raises concerns about such knowledge’s potential invisibilization or distortion during its systematization within scientific frameworks. For this reason, the publication of ethnobiological data can only be considered respectful if conducted ethically and with sensitivity to the sociocultural context from which the knowledge emerges. In practice, the community must be fully informed and actively involved in deciding whether a given piece of information should be published. Publishing without these considerations perpetuates epistemic inequalities, subordinating TEK/LEK systems to the paradigms of western science.

A key reflection arising from this discussion is that the publication of ethnobiological data must extend beyond simply adhering to ethical formalities. It is essential to promote epistemic justice, which involves valuing traditional knowledge and ensuring that communities actively participate in the process of knowledge construction. This requires a genuine co-production approach; wherein scientific and traditional epistemologies interact collaboratively and equitably without overshadowing or subordinating each other. Such collaboration may include agreements to publish or document knowledge in meaningful and relevant formats to the knowledge holders.

Moreover, we must acknowledge that science has both epistemological limits and ethical responsibilities when engaging with local knowledge. Publishing ethnobiological data without respecting these limits or prioritizing the rights and interests of the communities involved constitutes epistemic injustice perpetuating the historical marginalization of their knowledge systems. Therefore, the publication of ethnobiological data should be understood as a conditional tool—valid only when rigorous standards of ethics, equity, and respect are upheld.

A forward-looking approach to ethical ethnobiological research must not only critique existing challenges but also propose actionable strategies for improvement. To enhance ethical publishing practices, we advocate for the integration of ethical review frameworks that are both flexible and culturally sensitive, ensuring that institutional mechanisms support rather than hinder equitable collaborations. Additionally, greater investment in capacity-building programs for both researchers and IPLC/AMMC can foster a more just and inclusive research environment.

Scientific journals also play a pivotal role in this transformation. By developing editorial policies that explicitly require community involvement and co-authorship where appropriate, they can reinforce the ethical standards expected in ethnobiological research. Furthermore, traditional knowledge data should not be treated as taboo within the ethnobiological community. Its application in addressing environmental, social, and health-related challenges aligns with the interests of IPLC and AMMC. Rather than avoiding engagement with this knowledge due to ethical concerns, researchers should work to establish ethical and reciprocal partnerships that ensure fair benefit-sharing and proper recognition.

As we were writing this text, we came across an article by Zank et al. [24] that questions the future of the term “ethnobiology” and argues that its relevance may diminish as TEK/LEK systems gain visibility and recognition. While we acknowledge the importance of this discussion and the need for critical reflection on the colonial roots of ethnobiology, we respectfully disagree with their proposition to render the term obsolete. Instead, we argue that re-signifying “ethnobiology” is essential for fostering an inclusive and transformative scientific practice.

Ethnobiology, much like ethnology and anthropology, undeniably has a colonial legacy. These fields emerged within contexts where western science sought to dominate and categorize other knowledge systems, often relegating TEK/LEK to a subordinate position. However, precisely, this historical trajectory gives ethnobiology its unique potential to critically confront and dismantle these hierarchies. Rather than discarding the term, we advocate for a broader interpretation of “ethno”—one that no longer exclusively refers to IPLC and AMMC but instead encompasses the entirety of humanity and its diverse relationships with the natural world. Unsurprisingly, ethnobiological studies have expanded their thematic scope and engaged not only IPLC and AMMC but also a diverse range of human groups, reflecting the complex interactions between people and nature—a dynamic process central to the history of ethnobiology.

In this sense, ethnobiology is not a relic of the past but a dynamic and evolving discipline that has expanded beyond its colonial roots (see the fruitful discussion in – [4, 17]). Today, the field recognizes and integrates knowledge systems from all cultures, positioning them as equal contributors to a shared understanding of human-nature interactions. Proposing to eliminate the term, as Zank et al. [24] suggest, risks perpetuating another form of epistemicide—the erasure of a field that has been instrumental in advocating for the legitimacy and visibility of diverse knowledge systems.

An important point is that different types of knowledge, including scientific knowledge, have distinct characteristics. Recognizing these differences should not be seen to exclude or devalue non-scientific knowledge but as an acknowledgment that knowledge systems operate in different ways. From a philosophical perspective, scientific knowledge is built through specific methods of reasoning and structured research practices that distinguish it from the ways TEK/LEK is developed and maintained. Acknowledging these distinctions fosters meaningful interdisciplinary dialogues, allowing diverse knowledge systems to contribute to innovative solutions. Valuing epistemic diversity is essential. Moreover, defining a knowledge system as “scientific” is not necessarily beneficial, as it risks imposing a scientific label onto highly complex ways of knowing. This approach may homogenize diverse epistemologies rather than properly recognizing their intrinsic value.

True decolonization of ethnobiology and other disciplines cannot be achieved by merely rejecting terms or labels. Instead, it requires the transformation of practices, structures, and epistemologies. This process demands a constant, conscious, and active effort to deconstruct oppressive frameworks and foster equitable collaborations with IPLC and AMMC. Decolonization is not a one-time act but an ongoing commitment that must be embedded in the discipline’s methods, ethics, and epistemic frameworks.

Eliminating ethnobiology will not address the root causes of exclusion or inequity. On the contrary, it risks undermining decades of work to integrate diverse knowledge systems into global scientific discourse. The focus should instead be on re-signifying and strengthening ethnobiology as a platform for inclusivity and justice. By embracing its potential for transformation, ethnobiology can continue to play a vital role in addressing future challenges and fostering a collective, ancestral vision for sustainability.

## Data Availability

No datasets were generated or analyzed during the current study.

## Abbreviations

IPLC: Indigenous Peoples and Local Communities
AMMC: Afro-descendants and other marginalized, minority or minoritized communities
LEK: Local ecological knowledge
TEK: Traditional ecological knowledge
